# International Hahnemann Association

**Published:** 1881-04

**Authors:** 


### INTERNATIONAL HAHNEMANN ASSOCIATION.

The Bureau of Obstetrics, Diseases of Women and Children. The Chairman appoints the following:

H. N. Guernsey, M.D., Phila.; Gynæcology, its Principles and Treatment. L. B. Wells, M.D., Utica, N. Y.; Labor, its Abnormalities and their Treatment. W. H. Leonard, M.D., Minneapolis, Minn.; Uterine and Puerperal Hæmorrhage. C. Lippe, M.D., New York City; Diseases of Children, and their Treatment. T. F. Pomeroy, M.D., Chairman, Jersey City, subject not yet selected.

Bureau of Surgical Therapeutics.—J. G. Gilchrist, M.D., Detroit; J. B. Bell, M.D., Boston; J. C. Morgan, M.D., Phila.; R. R. Gregg, M.D., Buffalo; G. H. Carr, M.D., Whitehall, Michigan; C. F. Nichols, M.D., Boston; E. A. Ballard, M.D., Chairman, Chicago.

